# Syndromic surveillance systems to detect outbreaks of gastroenteritis in high-income countries: a scoping review

**DOI:** 10.1186/s12889-025-25329-w

**Published:** 2026-03-23

**Authors:** Faris Adel Mutawalli, Amalie Dyda, Satyamurthy Anuradha, Sheleigh Lawler, Phu Cong Do, Rehab Meckawy, Simon Reid

**Affiliations:** 1 School of Public Health, The University of Queensland, Herston, QLD Australia; 2 Health Information Technology Department, Faculty of Applied Studies, King Abdul Aziz University, Jeddah, Kingdom of Saudi Arabia; 3 Metro South Public Health Unit, Metro South Hospital and Health Service, Brisbane, QLD Australia; 4 Community Medicine Department, Faculty of Medicine, Alexandria University, Alexandria, Egypt

**Keywords:** Syndromic surveillance, Gastroenteritis outbreaks, High-income countries, Early outbreak detection, Over-the-counter medication, Telehealth, Web-based queries, School absenteeism, Health-seeking behaviour

## Abstract

**Background:**

Syndromic surveillance systems leverage non-diagnostic data sources to enable the early detection of infectious gastroenteritis outbreaks. However, their use in high-income countries (HICs) remains underexplored. This scoping review explores published literature on syndromic surveillance for gastroenteritis outbreaks in HICs, focusing on the types of data used, their effectiveness, and reported outcomes.

**Methods:**

A systematic search was conducted in PubMed, Embase, Scopus, and CINAHL for studies published from 2000 onwards. This scoping review followed the Preferred Reporting Items for Systematic Reviews and Meta-Analyses extension for Scoping Reviews (PRISMA-ScR) guidelines. Data extraction was conducted using a structured form adapted from the Joanna Briggs Institute (JBI) guidelines and the Centers for Disease Control and Prevention (CDC) framework for evaluating surveillance systems was used to systematically guide the extraction. Studies using diagnostic data or in-person healthcare visit data (e.g., GP or ED presentations) were excluded, in line with the syndromic surveillance purpose of early detection in HICs.

**Results:**

Ten studies met the inclusion criteria. The most common data sources were over-the-counter (OTC) medication sales (*n* = 5) and telehealth consultations (*n* = 4), followed by web-based queries (*n* = 2) and school absenteeism reports (*n* = 1). OTC and telehealth data provided timely, sensitive, and representative outbreak signals. However, challenges (e.g., seasonal stockpiling, demographic variability) affected OTC sales, while shifts in health-seeking behaviour influenced telehealth data reliability. Web-based queries showed early warning potential but were limited by geographic and demographic biases. School absenteeism data were useful for child-focused settings but had reporting limitations.

Most studies assessed timeliness and sensitivity, with limited evaluations of other key attributes such as representativeness, acceptability, and stability. No studies explicitly considered health-seeking behaviour as a rationale for data selection.

**Conclusions:**

This scoping review underscores the potential of syndromic surveillance for detecting gastroenteritis outbreaks in high-income countries. While most data sources could provide a signal for early outbreak detection, the findings highlight the need for more comprehensive evaluations, improved data representativeness, and the integration of health-seeking behaviour considerations in syndromic surveillance system design. These areas should be the focus of future research to enhance the effectiveness of syndromic surveillance for gastroenteritis in high-income countries.

**Supplementary Information:**

The online version contains supplementary material available at 10.1186/s12889-025-25329-w.

## Background

Each year, gastroenteritis causes approximately 1.45 million deaths and 89.5 million disability-adjusted life years (DALYs) globally [1]. Norovirus infection alone is associated with an annual economic burden of $4.2 billion in treatment costs and $60.3 billion in societal losses [1, 2]. The highest burden of gastroenteritis occurs in low- and middle-income countries (LMICs), where poor sanitation, water, and hygiene infrastructure exacerbate disease transmission [3, 4]. However, high-income countries (HICs) also experience significant economic impacts, primarily from illness-related morbidity and associated losses to productivity. For example, gastroenteritis causes approximately 21 million cases annually in France [5], 179 million estimated annual cases of acute gastroenteritis in the United State population, including approximately 1 million hospitalisations [6], up to 17 million estimated cases with 1 million general practice (GP) consultations annually in the United Kingdom [7], and an estimated 17.2 million gastroenteritis incidents across Australia from various pathogens [8]. This highlights the need for systems to rapidly detect and respond to prevent outbreaks and their associated costs [9–14].

Infectious gastroenteritis is caused by several pathogens that persist in diverse environments and spread through multiple mechanisms [15–17], resulting in outbreaks. Gastroenteritis outbreaks frequently occur in settings where close contact facilitates rapid disease transmission, such as aged-care facilities [18–20], schools and child-care centres [21, 22], camp facilities [23–25], healthcare settings [26], mass gatherings [27, 28] and cruise ships [29]. Given the substantial health and economic impact of gastroenteritis, robust public health surveillance systems are essential for early detection and response to mitigate the risks of outbreaks [30].

Public health surveillance is defined as “the ongoing, systematic collection, analysis, interpretation, and dissemination of data regarding a health-related event for use in public health action to reduce morbidity and mortality and to improve health ” [31]. The design and evaluation of surveillance systems take into account the nature of the health problem, the purpose of the system and its functional attributes [31]. We are thus able to describe how “useful” a surveillance system is based on its structure and function (simplicity and flexibility), the quality and acceptability of the data, its ability to accurately detect all outbreaks (sensitivity and predictive value positive), the potential reporting bias (representativeness), and its ability to function reliably in the necessary timeframe (stability and timeliness) [31].

Traditional communicable disease surveillance systems rely on mandatory reporting (notifications) of confirmed cases by laboratories for specified lists of pathogens [32]. These systems have significant drawbacks for the detection of acute outbreaks of gastroenteritis due to underreporting, poor representativeness, and reporting delays [33]. Indeed, gastroenteritis is often underreported in traditional surveillance systems because of the reliance on individuals seeking medical care for a condition that they may prefer or feel able to self-manage [2, 5, 34]. For example, a study found that only 30% of people experiencing gastrointestinal symptoms seek medical attention, with only 20% of these cases submitting stool samples for laboratory diagnosis [35]. In addition, these systems typically only include reports for a small fraction of the potential aetiological agents because the majority are not usually classified as notifiable, which excludes significant causes of morbidity, such as norovirus and *Giardia* [1, 36–39]. Moreover, traditional surveillance primarily relies on clinical diagnostic data, such as laboratory confirmations, which are not timely because of delays in submission/testing and the hierarchical structure of these systems [32, 40]. Such limitations complicate accurate burden estimation and hamper effective prevention and control efforts [15–17]. For example, during the 1993 cryptosporidiosis outbreak in Milwaukee, Wisconsin, earlier detection through effective surveillance systems could have potentially prevented up to 85% of the estimated 403,000 cases [41].

Syndromic surveillance systems have been developed as a complementary approach to routine systems, using real-time or near-real-time data from diverse health-related data sources such as over-the-counter medication (OTC) sales, telehealth consultations, and emergency department visits to detect patterns indicative of potential outbreaks [11, 32, 42]. Its primary goal is to facilitate the early detection of abnormal trends in disease patterns in a timely manner to public health agencies to detect and respond early to a potential threat [43]. According to the Centers for Disease Control and Prevention (CDC), syndromic surveillance is defined as a method used by public health staff as an investigational approach, which involves the use of automated data acquisition and generation of statistical alerts to monitor health-related data in real-time or near real-time to detect outbreaks earlier than traditional public health surveillance [44].

One of the historical uses of syndromic surveillance has been examined in a retrospective study that investigated the significance of eight data sources available during the 1993 Milwaukee cryptosporidiosis outbreak. The study highlighted that non-diagnostic data, such as over-the-counter medication sales, have the potential to detect the outbreak earlier than diagnostic data [45]. Currently, it is widely operationalised in high-income countries and recognised as a critical tool for early detection of infectious disease outbreaks. The UK Syndromic Surveillance Service (UK SSS) monitors more than 140 indicators derived from general practice in- and out-of-hours consultations, telehealth calls (NHS 111), and emergency department attendances to provide real-time situational awareness [46]. The Centers for Disease Control and Prevention operates the BioSense Platform as a cloud-based system to receive syndromic data from a participating facility with the Electronic Surveillance System for the Early Notification of Community based Epidemics (ESSENCE) functions as its integrated analytical tool for trend detection and visualisation to enhance health threat readiness and response [47].

It is important to note that syndromic surveillance is inherently data-driven, and its effectiveness depends on the quality, timeliness, and accuracy of the data collected [48]. It is also influenced by contextual factors such as available resources, the specific diseases or symptoms, and demographic and behavioural characteristics of the population under surveillance [49–51]. Despite advances in technology that have enabled the use of novel data [13, 32], there is limited information for gastroenteritis outbreaks in high-income settings, with an emphasis on the characteristics, performance, and rationale for the use of different data types. Multiple systematic reviews have evaluated the capability of syndromic surveillance for infectious disease outbreaks [50, 52, 53]. However, these have primarily emphasised detection capability rather than the influence of data type characteristics. While the findings demonstrate the potential of syndromic surveillance to provide early detection for infectious diseases, the literature shows inconsistencies in research on infectious gastroenteritis, with a predominant focus on respiratory syndromes. This leaves a gap in understanding the influence of the data type used in syndromic surveillance for gastroenteritis outbreaks in high-income countries.

Therefore, the research fills this knowledge gap by exploring the types of data used in syndromic surveillance for the detection of infectious gastroenteritis at its early stages in high-income countries, while highlighting the characteristics of the system, the type of data used and its reported advantages and disadvantages. Understanding how different data types have been used and their effectiveness will help identify best practices and inform future research. Additionally, given the impact of the health-seeking behaviour on the data type, this review will also identify the rationale behind the choice of data types across studies, providing insights into current practices and identifying research gaps to guide future studies.

## Methods

### Study design

This scoping review followed the Preferred Reporting Items for Systematic Reviews and Meta-Analyses extension for Scoping Reviews (PRISMA-ScR) guidelines [54].

### Information sources and search strategy

In July 2023, a comprehensive literature search was conducted across four electronic databases: PubMed, Embase, Scopus, and CINAHL. The search was restricted to studies published from 2000, as bibliometric analysis showed an increase in syndromic surveillance published articles since 2005 [55], and the recent advancements in digital tools, real-time analytics, and electronic health records, over the past two decades, have significantly transformed syndromic surveillance systems, making more recent studies relevant for understanding current capabilities and practices [13].

The search strategy combined keywords and MeSH terms related to syndromic surveillance and gastroenteritis. An experienced librarian provided guidance in refining the strategy to maintain consistency across databases. The included search terms encompassing concepts such as “early warning systems,” “health indicator surveillance,” and “prodrome surveillance” to capture studies that may not explicitly use the term “syndromic surveillance.”

The search terms included combinations such as *(“syndromic surveillance” AND data sources like “telehealth*,*” “over-the-counter drugs”) AND (“gastroenteritis” OR symptoms like “vomiting*,*” “diarrhoea*,*” or pathogens like “Norovirus*,*” “Cryptosporidium”).*

An updated search was conducted in September 2024, but no additional studies found to meet the inclusion criteria. The detailed search strategies for each database are provided in Supplementary Materials, Table S1.

### Study selection

The first author (FM) conducted the initial search, exported results, and removed duplicates using EndNote 20 (both automatically and manually). Two independent reviewers (FM and PD) screened titles and abstracts based on the eligibility criteria. Full-text reviews were conducted by FM and PD, with any disagreements resolved through team discussion.

### Eligibility criteria

Studies that evaluated and validated routine syndromic surveillance systems for the early detection of infectious gastroenteritis outbreaks in HICs were included. High-income countries were defined based on the Organisation for Economic Co-operation and Development (OECD), as they are generally reflective of high-income countries [56, 57].

Only systems implemented or planned for implementation at the local or municipal level were included to avoid delays associated with the hierarchical structure and data collection processes of syndromic surveillance inherent in broader national-level systems. These systems were required to operate within specific geographic regions, such as cities, towns, or districts, rather than at the national or broader regional level.

We applied the following exclusion criteria:

- Studies using data that required in-person healthcare visits, including emergency department (ED) and Healthcare personnel visits.
- Studies on syndromic surveillance for specific events (e.g., mass gatherings, natural disasters).
- Research unrelated to outbreak detection (e.g., vaccine uptake monitoring).
- Simulation-based studies.
- Research focused on non-infectious causes of gastroenteritis (e.g., food allergies).
- Non-English publications, commentaries, reviews, posters, editorials, and preprint articles.
- Studies involving animal or non-human subjects.

### Data extraction

Data extraction was conducted using a structured form adapted from the Joanna Briggs Institute (JBI) guidelines. Data were extracted across three primary areas:

- Study Characteristics: Authors, publication year, study design.
- System Description: Country, population under surveillance, data sources, data type, Syndromes or symptoms monitored, reference data and detection methods.
- Data Type Findings: Rationale for data type selection and associated outcome.

Data on the performance attributes (Task D) described in the Centers for Disease Control and Prevention (CDC) framework for evaluating surveillance systems (CDC framework) [31] were extracted. They include: Simplicity, Flexibility, Data Quality, Acceptability, Sensitivity, Predictive Value Positive, Representativeness, Timeliness, and Stability [31]. The attributes were used to systematically organise the reported outcomes associated with different data types in syndromic surveillance. This approach allowed us to extract data consistently and identify whether the studies formally evaluated these performance attributes. We used three classifications for each attribute based on study findings.

- Evaluated: Formally assessed in the study.
- Reported: Attribute was mentioned but not formally analysed.
- NR: Not Reported.

The data extraction fields were iteratively refined as new relevant information emerged across the studies.

### Data synthesis

Data were synthesised into a summary of the included studies and a narrative summary of outcomes, organised by data type. Due to the heterogeneity of the studies, direct comparisons between data types were not possible.

## Results

**Fig. 1:**
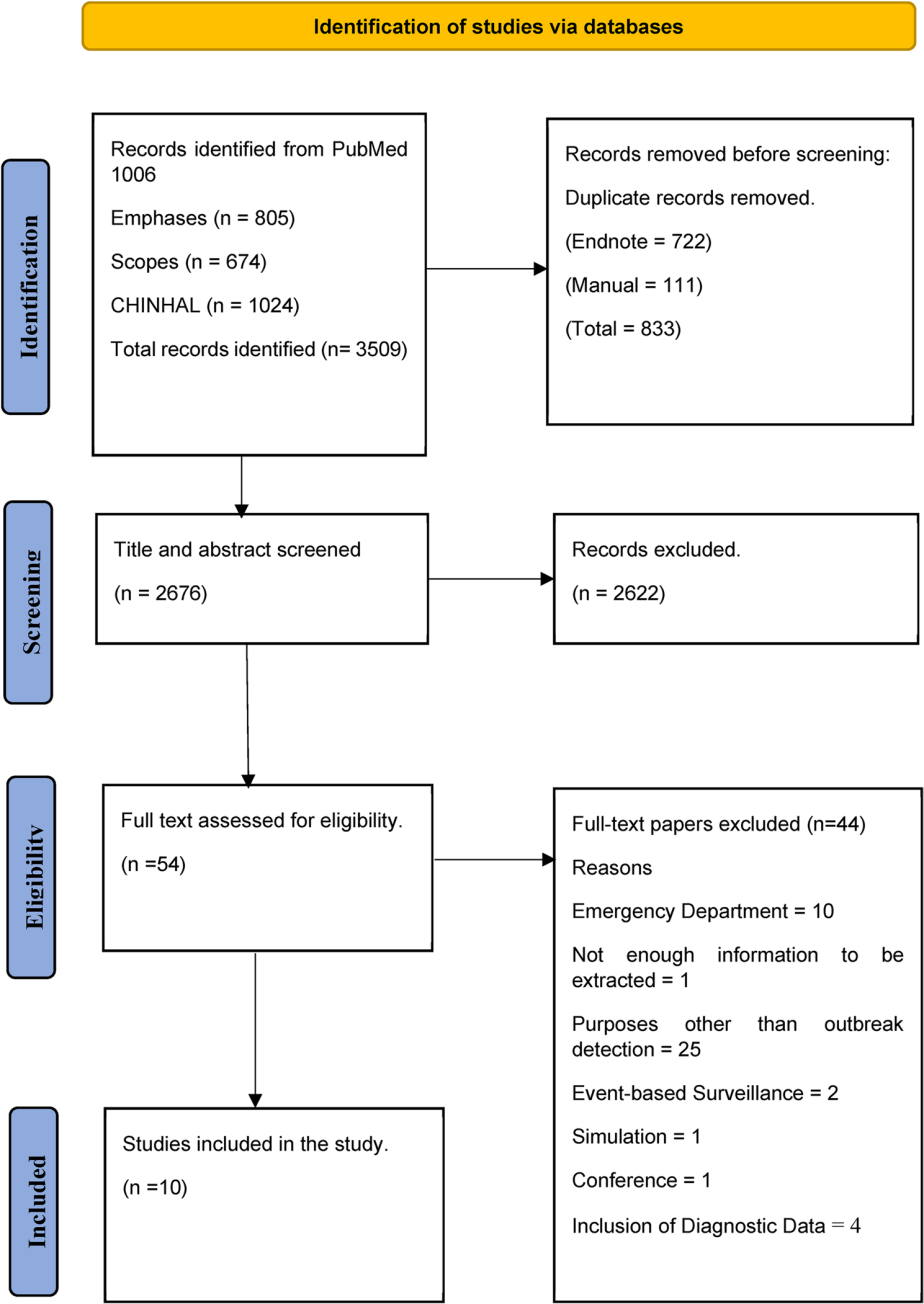
PRISMA flow diagram

The search strategy identified a total of 3,509 articles. After removing 833 duplicates, we screened the titles and abstracts of 2,676 articles and excluded 2,622 based on relevance. Of the remaining 54 studies, a further 44 articles were excluded because they did not report on the use of syndromic surveillance for early detection of gastroenteritis outbreaks in high-income countries. This process resulted in the final inclusion of 10 studies for this review. Reasons for exclusion are in Fig. 1.

### Study characteristics

The ten included studies were conducted across various countries, including the United States [58], Canada [10, 59–61], Sweden [62, 63], France [64], Japan [65], and England [51]. These studies were published between 2003 and 2022, with three studies conducted before 2014 [10, 58, 59] and seven were conducted afterwards [51, 60–65]. Eight studies employed retrospective analysis and two studies used both retrospective and prospective analysis in their evaluation [60, 64]. One study combined retrospective analysis (2008–2012) with prospective monitoring (2013–2014) to compare historical data with real-time observations [64], while the other applied retrospective analysis at the local level and integrated prospective analysis at the national level [60].

Table 1 summarises the key characteristics of the included studies and their surveillance systems. All included studies primarily evaluated the effectiveness of syndromic surveillance systems in detecting gastroenteritis outbreaks. In addition, two studies also included data on respiratory illness [58, 60], and one study assessed the added value of the syndromic surveillance in providing early warning signals for a number of disease syndromes, including gastroenteritis [65].

**Table 1:** Summary of included studies and their syndromic surveillance systems

Author (Year)	Country	Population Under Surveillance	Study Design	Data Type	Rationale for Data Selection	Reported Capability of Detection	Reported Public Health Response
Hogan et al. (2003)	United States	Paediatric population	Retrospective	OTC Medication	Availability, Biological plausibility	Strong correlation (0.90) with hospital diagnoses. Sales data detected outbreaks 1.7 weeks earlier, with the EWMA algorithm confirming early detection by 2.4 weeks on average. Data covered 90% of the market, but variability across outbreaks and potential false positives due to unrelated sales spikes were noted.	Not reported
Edge et al. (2004)	Canada	General population	Retrospective	OTC Medication	Availability, Prior study evidence	Detected outbreaks timely. MA and CUSUM plots identified two periods of increased sales (Dec 1999, May 2000) corresponding to gastroenteritis outbreaks. OTC data provided earlier outbreak signals than traditional surveillance methods. However, data limitations include seasonal fluctuations, promotional sales, and variability across sites, requiring adjustments to detection algorithms.	Not reported
Caudle et al. (2009)	Canada	General population	Retrospective	Telehealth	Availability, Prior study evidence	High correlation with ED visits (0.90). Spearman correlation (0.90, *p* < 0.0001) between Telehealth call volumes and NACRS (ED) data. Vomiting and diarrhoea were the most reported symptoms, with vomiting (59%) more common in children (0–4 years). Real-time 24/7/365 centralised electronic data collection enhances timeliness and efficiency.	Not reported
Andersson et al. (2014)	Sweden	General population	Retrospective	1-Telehealth 2-OTC 3-Web Queries	Availability, Prior study evidence	1-Telehealth: Most effective for detecting point-source outbreaks, with clear signals for outbreaks exceeding 1,000 cases. 2-OTC Sales: Captured the two largest diarrhoea outbreaks but had extreme values that did not always align with known outbreaks. 3-Web Queries: Lacked sufficient geographic resolution; no outbreak signals detected.	Not reported
Edelstein et al. (2014)	Sweden	General population	Retrospective	Web Queries	Availability, Prior study evidence	Detected outbreaks 2–3 weeks earlier than laboratory confirmations. Search trends showed strong correlations with confirmed cases. Early warnings were observed in multiple outbreak seasons. Representativeness was limited, as users were predominantly from specific demographic groups. Limitations: Data may be influenced by non-outbreak-related searches, and anonymity prevents direct linkage to confirmed cases.	Not reported
Pivette et al. (2014)	France	General population	Retrospective & Prospective	OTC & Prescribed Medication	Availability, Prior study evidence	OTC sales detected outbreaks 2.25 weeks earlier than general practitioner (GP) reports. The system demonstrated high sensitivity and reliability, detecting all seasonal epidemics without false alerts. Daily data collection enabled real-time monitoring, improving the timeliness of public health responses. Prescribed medications were more closely correlated with reported cases but showed delayed detection due to consultation requirements. Limitations included non-specificity of certain medications, stockpiling behaviours, and external factors like advertising influencing sales patterns.	Not reported
Muchaal et al. (2015)	Canada	General population	Retrospective & Prospective	OTC Medication	Availability, Prior study evidence	Limited effectiveness for gastrointestinal outbreaks. No clear correlation was found between gastrointestinal OTC sales and outbreak occurrences. Pharmacy-based surveillance was less effective for gastrointestinal outbreaks compared to respiratory illness surveillance. A key limitation was the exclusion of healthcare institution data, particularly from long-term care facilities, where the majority of outbreaks occurred, affecting the accuracy of detection.	Not reported
Tanabe et al. (2019)	Japan	Children < 18 years	Retrospective	Absenteeism	Availability, Prior study evidence	Absenteeism data demonstrated a 78.8% success rate in preventing outbreak clusters, with daily reporting allowing for real-time monitoring and early public health responses. Lag models showed that absenteeism data could provide early warning signals, capturing cases that may not present to healthcare facilities. However, limitations included unknown reasons for school absences, variability in reporting, and the impact of alternative health-seeking behaviours (e.g., online self-care advice).	Reported – The system initiated 85 public health calls, 70 of which addressed diarrhoea, vomiting, or gastroenteritis (GI). These actions reportedly prevented 67 GI-related outbreak clusters (95.7% avoidance).
Hughes et al. (2019)	Canada	General population	Retrospective	Telehealth	Availability, Prior study evidence	Telehealth data provided a 2-week lead time for outbreak detection compared to laboratory-confirmed cases. The study highlighted that telehealth data captured individuals who did not present to healthcare services, making it a valuable real-time surveillance tool. However, limitations included shifts in health-seeking behaviour, reduced telehealth utilization, and a lack of detailed information on physician testing decisions.	Not reported
Donaldson et al. (2022)	England	General population	Retrospective	Telehealth	Availability, Prior study evidence	The system detected outbreaks earlier than traditional surveillance methods, providing an early warning of seasonal illness trends. NHS 111 calls provided near real-time data on vomiting and diarrhoea symptoms, allowing for timely outbreak detection. However, challenges included reporting bias, seasonal gaps in school data, and difficulty attributing outbreaks to specific pathogens. The study highlighted that self-reported symptoms may lead to overreporting of mild cases, while voluntary reporting from schools and care homes could result in underreporting. Despite these limitations, the system proved valuable for tracking outbreaks across multiple settings, including care homes, hospitals, and schools.	Not reported

### Description of data used in syndromic surveillance

#### Syndromic surveillance using over-the-counter medication

Five studies employed over-the-counter medication sales data to track gastroenteritis trends [58–60, 62, 64] among the general population, with one focusing on the paediatric population [58]. Of these, four studies collected data from established sources that collected sales data regularly from pharmacies daily [60, 62, 64], and weekly at the point of sale from pharmacies, grocery stores and mass retailers (e.g., Kmart) aggregated by store zip code [58]. Only one study collected data from two local pharmacies [59].

Different products were monitored across the studies. This included electrolyte product sales to detect dehydration-related symptoms [58], anti-diarrheal OTC medications [62], weekly sales of anti-diarrheal and anti-nauseant products [59] and gastrointestinal product sales data [60]. One study monitored both prescribed and OTC medications such as antiemetics, anti-diarrheal, and oral rehydration solutions [64].

### Syndromic surveillance using telehealth data

Four studies used telehealth data for syndromic gastroenteritis surveillance [10, 51, 61, 62]. All studies focused on the general population, collecting data from established public health telehealth services. The primary symptoms monitored included diarrhoea, vomiting, and other gastrointestinal symptoms, with specific data fields varying by study. For instance, Caudle et al. [10] monitored telehealth call volumes for symptoms such as unusual stool colour and blood in stools in paediatric cases. Andersson et al. [62] tracked symptoms such as nausea, vomiting, and stomach pain, while Donaldson et al. [51] focused on vomiting and diarrhoea alongside consultations for gastrointestinal conditions recorded in general medical practices.

### Syndromic surveillance using Web-Based query data

Two studies utilised web search query data to conduct real-time monitoring of gastroenteritis symptoms [62, 63]. Both studies were conducted in Sweden, collecting data from public health portals where search queries were considered indicators of gastrointestinal symptoms within the general population. The Websök system tracked real-time searches for terms related to vomiting, diarrhoea, and winter vomiting disease, aiming to detect seasonal patterns and potential local outbreaks [63]. Additionally, data were collected from Vårdguiden, the Stockholm County Council’s health information website, which captured queries about symptoms such as nausea, vomiting, diarrhoea, stomach pain, and general gastrointestinal illness [62].

### Syndromic surveillance using school absenteeism data

One study conducted in Japan utilised school absenteeism data for syndromic surveillance, focusing on children younger than 18 years attending nursery and primary schools [65]. The Nursery School Absenteeism Surveillance System (N-SASSy) collected absenteeism reports from school nurses and teachers, noting absences due to symptoms such as diarrhoea and vomiting. The system uses historical patterns within the system to establish baseline levels, identifying deviations that might indicate an outbreak.

### Method of analysis and reference data of the included studies

The methods of analysis described in these studies were applied for two primary purposes: detecting outbreaks and validating the reliability of syndromic surveillance data. Time-series analyses were employed in five studies [10, 51, 58, 59, 64] to monitor trends and detect anomalies. Methods included Exponentially Weighted Moving Average (EWMA) [58], CUSUM and Moving Average [59], time-series analysis of telehealth data [10], Serfling-type periodic regression [64], and breakpoint analysis [51]. Threshold-based methods were reported in two studies [62, 65], including weak (3 SD) and strong (5 SD) deviation classification [59] and the Early Aberration Reporting System (EARS) C1 indicator, which flagged deviations exceeding three standard deviations from a seven-day baseline for absenteeism monitoring [65]. Additionally, one study applied the Shewhart Control Chart for anomaly detection in telehealth vomiting calls [61].

Validation was achieved through correlation, regression, and sensitivity and specificity analyses. Correlation analyses were used in four studies [10, 58, 63, 64], including Spearman correlation [10] and cross-correlation analysis [63, 64]. Regression models were employed in two studies [61, 64], with Pivette et al. [64] prospectively validating the system using periodic regression model and Hughes et al. [61] applying negative binomial regression to validate telehealth calls. Sensitivity and specificity were assessed in two studies [60, 62]. Descriptive and temporal analyses were used in two studies [51, 60], with Muchaal et al. [60] comparing OTC sales to confirmed outbreaks and Donaldson et al. [51] applying lagged correlation, partial correlation, and breakpoint analysis to validate early outbreak signals.

Reference data used to validate OTC sales data include hospital admissions data using ICD-9-CM codes [58], ED visits [59], public health outbreak reports [60], and general practitioner networks (French Sentinel Network) [64]. For telehealth data, the studies used data from ED visits [10], known waterborne and foodborne outbreaks [62], laboratory-confirmed data [61], and both laboratory-confirmed norovirus cases and outbreaks reported in care homes and hospitals [51]. Web search queries were validated against known waterborne and foodborne outbreaks [62], and laboratory-confirmed cases of norovirus [63].

### Reported findings by data type

The review identified four data types across the included studies. The most commonly used data type was OTC, reported in five studies [58–60, 62, 64], followed by telehealth data which appeared in four studies [10, 51, 61, 62]. The least common type of data was web-based quarries which was reported twice [62, 63], and absenteeism data was used only in one study [65]. Overall, most studies reported the capability of syndromic surveillance to provide early detection of gastroenteritis outbreaks, using these data types. However, each data type demonstrated strengths in timeliness and sensitivity, but limitations in representativeness and data quality were consistently noted.

Table 2 provides a summary of the performance attributes in the included studies. None of the studies conducted a comprehensive, formal evaluation of all the performance attributes outlined in the CDC framework. Three attributes were reported across all the included studies: timeliness, which was evaluated in three studies [62–64], while sensitivity [62, 64] and representativeness [62, 63] were each evaluated in two studies. Data quality was reported in six studies and evaluated in two [63, 64], and simplicity and flexibility were reported in seven and six studies, respectively. The lowest reported attributes include acceptability, mentioned in two studies and evaluated in one [63], and stability, reported only twice. Notably, none of the studies reported a positive predictive value.

**Table 2:** Performance attributes summary in the included studies

Authors & Year	Simp	Flex	DQ	Accept	Sens	PV+	Rep	Time	Stab	Formal Evaluation sum
Hogan et al. (2003) (54)	R	NR	R	NR	R	NR	R	R	NR	No
Edge et al. (2004) (55)	NR	NR	R	NR	R	NR	R	R	NR	No
Caudle et al. (2009) (10)	R	NR	R	NR	R	NR	R	R	R	No
Andersson et al. (2014)(58)	R	R	R	NR	EV	NR	EV	EV	NR	Sens-Time-Rep
Edelstein et al. (2014) (59)	R	R	EV	EV	R	NR	EV	EV	NR	DQ-Time-Rep-Accept
Pivette et al. (2014) (60)	R	R	EV	NR	EV	NR	R	EV	NR	DQ-Sens-Time
Muchaal et al. (2015) (56)	R	R	R	R	R	NR	R.	R	NR	No
Tanabe et al. (2019)(61)	R	R	NR	R	R	NR	R	R	NR	No
Hughes et al. (2019) (57)	NR	NR	R	NR	R	NR	R	R	NR	No
Donaldson et al. (2022) (62)	NR	R	NR	NR	R	NR	R	R	R	No

Evaluated (EV) Formally evaluated in the study, *R *Reported, Attribute was mentioned but not formally analysed, *NR* Not Reported, Not Reported, *Simp* Simplicity, *Flex *Flexibility, *DQ *Data Quality, *Accept* Acceptability, *Sens *Sensitivity, *PV+* Predictive Value Positive, *Rep* Representativeness, *Tim *Timeliness, *Stab *Stability

### Over-the-counter medication sales data

Over-the-counter medication sales data displayed timeliness and sensitivity, which allowed for more immediate public health responses in several studies. All five studies consistently reported that OTC was timelier compared to traditional data [58–60, 62, 64]. For instance, one study reported that OTC enabled outbreak detection up to 1.7 weeks earlier than hospital data with outbreaks detected 2.4 weeks earlier using the Exponentially Weighted Moving Average (EWMA) algorithm [58]. Another study compared the timeliness of OTC data with general practitioner (GP)-reported cases and prescribed drug sales. The findings indicated that OTC data could detect outbreaks approximately 2.25 weeks earlier than GP-reported cases, whereas prescribed drug sales provided a mean lead time of only 0.6 weeks, reflecting their closer alignment with medical consultations but underscoring their timeliness compared to OTC [64].

Among these studies, three demonstrate the ability of the data to detect (sensitivity) gastroenteritis outbreaks [58, 59, 64]. One study reported that OTC data allowed the detection of 12 out of 18 outbreaks (67%) and reported a strong correlation between electrolyte sales and hospital diagnoses [58]. However, one study reported partial capability [62], highlighting that OTC sales data only captured the two largest diarrhoea outbreaks among the four known outbreaks, resulting in extreme values that did not match known outbreaks since symptoms were varied and more transient for the other two outbreaks. Only one study found OTC sales data less effective, where no clear correlation existed between sales and confirmed outbreaks, suggesting regional and demographic differences in detection efficacy [60].

Representativeness was reported across the five studies, with varying strengths and limitations. One study reported that OTC data was broadly representative in terms of place, covering over 90% of the market in the regions studied [58]. Similarly, another study reported that OTC data spanned over 85% of health regions across the country [60]. However, the study noted limitations in representativeness in areas lacking comprehensive healthcare institution OTC data, where most of the outbreaks in this area were reported from settings such as healthcare institutions and long-term care facilities. Notably, one study emphasised the significance of OTC sales data, particularly in contexts where individuals are more likely to self-medicate rather than seek formal healthcare services [59]. Potential demographic variation in medication usage were acknowledged as a limitation in another study [64].

Challenges in data quality were noted, as external factors such as promotional sales, seasonal patterns, and stockpiling behaviours introduced variability, complicating accurate detection [62, 64]. Simplicity was highlighted in four studies [58, 60, 62, 64], where automated point-of-sale data collection facilitated integration into surveillance systems. The flexibility of the data reported in three studies suggests that the OTC data could be adapted for other infectious diseases and monitored multiple product types highlighting its ability to adjust to different public health needs [60, 62, 64]. Acceptability was addressed through a stakeholder survey with 87% of respondents identifying that pharmacy information was important in their jurisdiction [60].

### Telehealth data

Four studies evaluated telehealth data, each addressing its capability to detect gastroenteritis outbreaks [10, 51, 61, 62]. One study found that telehealth data could accurately detect increases in disease and reported a high correlation between Telehealth call volumes and emergency department visits for gastrointestinal cases [10], while another study found that telehealth was particularly sensitive to large-scale illness outbreaks [62].

Representativeness was found to be a strong feature of telehealth data across the four studies, noting that these systems provide broad coverage, including in underserved rural areas. Two studies reported that telehealth data captured illness trends among individuals who might not seek traditional healthcare or receive laboratory confirmation [10, 61], with one highlighting the availability of the telehealth system to all residents 24/7, providing broad population coverage [61]. In addition, one study indicated that telehealth data provided the most representative and reliable signals compared to web queries and OTC sales data. However, two studies cautioned that shifts in health-seeking behaviours, such as increased internet-based self-care, could impact representativeness over time [10, 61]. The type of symptoms monitored also influenced data representativeness, with one study noting that nearly half of telehealth calls were from individuals experiencing vomiting [51].

Timeliness was observed to be a key advantage of telehealth data in these studies compared to traditional data. Hughes et al. [61] highlighted the timeliness of telehealth, noting a 2-week lead time for norovirus detection compared to laboratory data. One study noted a strong timeliness of telehealth data with comparison to other data such as OTC or Web Queries. In some cases, telehealth data detected outbreak signals weeks or even months before public recognition, underscoring its potential for early detection [62].

The simplicity of telehealth was highlighted in one study as centralised, real-time data collection, which facilitated the data-gathering process for public health monitoring [10]. Flexibility was reported in two studies [51, 62]. Both studies highlighted the flexibility of telehealth systems, illustrating the data’s ability to monitor various illnesses across diverse settings such as schools, hospitals, and care homes.

Data quality was reported in three studies showing a good level of data quality [10, 61, 62]. One study supported data quality through the use of standardised computerised decision trees, suggested a reliable framework for consistent data capture, though potential coding limitations were noted [10]. Another study highlighted the need for statistical adjustments to account for trends and seasonal effects, such as holiday variations in call volumes, to maintain data consistency and reliability in outbreak detection [61]. The stability of telehealth data was highlighted by Caudle et al., who noted that Telehealth Ontario’s continuous 24/7 data collection supports its role as a reliable long-term public health monitoring tool [10]. Overall, telehealth data proved to be a timely, sensitive, and adaptable resource for detecting outbreaks, though evolving health behaviours may affect its future consistency and reach.

### Web-based queries

Two studies evaluated the role of web-based queries in outbreak surveillance, reporting mixed results in their detection capabilities [62, 63]. One study confirmed the effectiveness of the system, noting that web query data detected norovirus outbreaks 2–3 weeks earlier than laboratory data, providing timely signals for early intervention [63]. The same study showed strong correlations with laboratory notifications for norovirus cases with “Winter vomiting disease” and “Vomiting,” underscoring its accuracy [63]. However, while the previous information can indicate adequate sensitivity and timeliness of web query data, the other study identified a significant limitation in the sensitivity of web queries, as no detectable signals for gastroenteritis outbreaks were found [62].

Representativeness was identified as a limitation in both studies. One study found that system users were more likely to be female, university-educated, aged 31–65, and predominantly based in Stockholm County, where 46% of users resided despite the county representing only 22% of Sweden’s population [63]. Another study noted that web queries lacked geographical resolution, which limited the broader applicability of the data across regions [62].

Simplicity was a prominent feature in both studies where one reported that the use of the data required minimal infrastructure, making it a low-cost and scalable solution for public health monitoring. Flexibility was also highlighted, with Websök system initially designed for influenza-like illness surveillance and later adapted for norovirus detection, demonstrating adaptability to emerging health needs [63].

Acceptability was evaluated in one study [63], with surveys indicating that 65% of infection control teams believed Websök could support infection control planning, and 54% considered it as reliable as laboratory data. Data quality was rated to be high in the same study [63], given the strong correlations with laboratory data, but the anonymity of search data posed limitations in directly linking searches to confirmed cases.

### School absenteeism data

Only Tanabe et al. [65], evaluated school absenteeism data for detecting outbreaks, reporting its detection capability for gastroenteritis and emphasising its timeliness through daily data collection, enabling real-time monitoring and rapid intervention. The study reported a 78.8% success rate in preventing outbreaks via early detection, underscoring the system’s utility in supporting timely public health responses. Representativeness was indicated by the coverage of 60% of schools and 40% of nurseries in Japan, providing a comprehensive sample of the child population. Flexibility was demonstrated through the system’s adaptability across various infectious diseases and its potential for application during large-scale events. Additionally, the system’s simplicity was evident in its computerised network, which facilitated the efficient sharing of health information across nursery schools and schools among relevant organisations and individuals.

Limitations were noted in sensitivity, as the system relied on caregiver-reported symptoms, leading to potential underreporting of mild or asymptomatic cases. Furthermore, unlike formal schools, nursery schools were not legally required to adhere to attendance prohibition rules for infectious diseases. This makes data from these centres dependent on voluntary caregiver reporting and affects the sensitivity and representativeness of health data collected. Additionally, non-standardised data entry times across schools impacted stability and consistency in detection, reducing reliability.

### Rationale for data selection across studies

Various considerations influenced the selection of data types for syndromic surveillance in these studies. Availability was a key factor in all ten studies, ensuring accessible and routinely collected data. Additionally, prior research evidence indicating the usefulness of this data to provide earlier warnings than traditional methods influenced their selection in all the included studies. Biological plausibility was explicitly cited in one study [58], which referenced the American Academy of Paediatrics’ recommendation for oral rehydration therapy with electrolyte products for children aged 1 month to 5 years as a justification for using electrolyte sales data in syndromic surveillance. However, none of the included studies explicitly considered the health-seeking behaviour of the population under surveillance as a rationale for data selection during study design. See Table 1.

## Discussion

This review demonstrates that non-diagnostic data, especially those that do not require in-person healthcare visits, have the potential to support early outbreak detection for syndromic surveillance of gastroenteritis outbreaks in HICs. Despite the observed potential advantages of syndromic surveillance, only 10 studies were identified, making the evidence base limited and not providing conclusive findings. The limited number of studies identified may reflect both the relatively low uptake of syndromic surveillance approaches for the early detection of gastroenteritis outbreaks and the specific eligibility criteria applied in this review.

Syndromic surveillance is designed to capture early indicators of illness using non-diagnostic data, which can precede clinical pre-diagnostic data that require healthcare visits and diagnostic data, such as laboratory confirmation. Surveillance data can be classified based on the point of origin along the illness timeline into “pre-clinical” data, which refer to behavioural signals that occur before any healthcare contact, such as over-the-counter medication sales, web searches, and absenteeism. “Clinical pre-diagnostic” data is generated once care is sought but before diagnostic confirmation, including telehealth consultations and emergency department chief complaints. “Diagnostic” data relies on laboratory-confirmed results or epidemiological case interviews and are typically less timely but more specific [11, 42]. The classification system makes a trade-off between timeliness and specificity because pre-clinical and clinical pre-diagnostic data offer greater timeliness, while diagnostic data provide higher specificity [11, 42]. Given the focus of syndromic surveillance on early detection, studies using diagnostic data were excluded from this review.

Furthermore, gastroenteritis is often self-limiting, and many individuals do not seek medical care. A national survey from 2008 to 2009 found that only 13.4% of individuals with gastroenteritis symptoms consulted a healthcare provider, with an even smaller proportion submitting stool samples for testing [66]. Another study reported that 30% sought care, but only 20% of those provided a stool sample [35], and more recent estimates suggest 19% of gastroenteritis cases result in a medical visit [39]. Given this low rate of healthcare utilisation, surveillance systems reliant on in-person visits may substantially underestimate the true burden of illness. Consequently, given the low healthcare utilisation rate for gastroenteritis, studies relying primarily on data requiring in-person healthcare visits were excluded.

Moreover, evidence suggests that ED-based syndromic surveillance is limited in its effectiveness for detecting gastroenteritis outbreaks, particularly in high-income countries, where mild cases are less likely to result in emergency care visits [67–69]. While ED surveillance systems have been useful for monitoring pandemic influenza, mass gatherings, and public health emergencies, they have shown limited capability in identifying localised gastroenteritis outbreaks [67, 69, 70]. This is primarily due to the low proportion of individuals with gastroenteritis seeking emergency care [69], the underrepresentation of mild cases in ED records [67, 70], and challenges related to data completeness and timeliness in ED-based surveillance [68, 70]. As a result, studies incorporating ED data in syndromic surveillance systems for gastroenteritis were excluded.

This review suggests that non-diagnostic data, especially those that do not require in-person healthcare visits, may be better suited for syndromic surveillance of gastroenteritis. These data types may offer a timelier and more representative picture of community illness, especially for conditions with low care-seeking behaviour. Given these findings, we recommend prioritising surveillance systems that incorporate data sources like telehealth, OTC and web search trends, which are both scalable and appropriate for capturing self-limiting conditions like gastroenteritis.

The heterogeneity of syndromic surveillance systems observed in these studies was evident across three key dimensions: data collection methods, symptoms monitored, and detection methodologies. While this heterogeneity may reflect the adaptability of syndromic surveillance, allowing for flexibility and adaptation to local needs, it also presents challenges for standardisation and cross-system comparisons. This is important because standard surveillance and outbreak investigation approaches require a clear, consistent case definition to identify at-risk populations and guide timely public health responses. As outlined in guidance such as the CDC’s Field Epidemiology Manual, key steps in outbreak investigation include identifying and defining cases by person, place, and time [71]. Thus, when data collection methods, symptom definitions, and detection algorithms vary, it becomes difficult to apply these key dimensions consistently from one system to another. While the limited number of studies restricts generalisability, this review nevertheless suggests that stakeholders interested in adopting such systems to carefully address this heterogeneity in system design and evaluation to ensure effective implementation and public health benefit in the target area.

For example, over-the-counter medication sales data and telehealth data provided the most useful data for syndromic surveillance, with more timely, sensitive, and representative outbreak detection. However, there were advantages and disadvantages associated with each system that need to be addressed during the design of the syndromic surveillance system. Over-the-counter medication sales data demonstrated notable timeliness, often providing early warning signals weeks ahead of traditional data sources. Its simplicity, flexibility, and availability enhance its appeal as a surveillance tool. However, data quality challenges, such as promotional sales and seasonal stockpiling, introduce variability that complicates outbreak detection [62, 64], Additionally, regional and demographic differences impact representativeness, with some studies noting limitations in areas where self-medication behaviours or healthcare infrastructure vary significantly [60, 64]. Representativeness and sensitivity may be improved by expanding OTC surveillance beyond pharmacies to include sales data from a broader range of retail settings, such as grocery stores and supermarkets [58]. Furthermore, incorporating OTC data from healthcare institutions, such as long-term care facilities, which may source medications differently than the general public, could enhance surveillance coverage and improve outbreak detection accuracy [60].

Telehealth data proved to be sensitive and timely data for detecting gastroenteritis outbreaks. Its broad coverage, particularly in underserved areas, enhances its representativeness, and its adaptability allows monitoring across various health contexts. However, the reliability of telehealth data could be negatively impacted by the shifts in health-seeking behaviours, such as increased reliance on internet-based self-care [10, 61]. Addressing these shifts is crucial to maintain the utility of telehealth data in syndromic surveillance systems. This requires new analytical approaches that incorporate contextual factors that influence these behavioural trends, such as machine learning, which can dynamically adapt to changing data patterns by continuously learning from recent inputs. For example, recent studies have shown that combining machine learning with dynamic systems modelling can enhance the early detection of respiratory outbreaks in primary healthcare data [72]. In addition, Lake et al. [73] demonstrated that machine learning techniques can complement aberration detection methods such as RAMMIE, Farrington, or EARS by interpreting alarms and adapting to evolving data streams, leading to better decision making. However, the authors emphasised that human oversight remains critical for contextual understanding and system responsiveness [73].

The simplicity, flexibility, and acceptability of web-based query data support its integration into syndromic surveillance systems because it has been shown to provide a signal to detect outbreaks weeks earlier than laboratory data. However, this data source is limited by geographical resolution and demographic biases, as users tend to belong to specific population groups (e.g., urban, highly educated, Female) [62, 63]. For example, without geographical resolution, the system may detect an increase in a particular symptom, indicating a potential outbreak, but it would be difficult to localise the source or determine which specific regions are affected [63]. This limits the ability of public health authorities to implement targeted interventions or allocate resources effectively. These representativeness limitations suggest that web-based query data is most effectively used as a complementary tool rather than a primary data source for syndromic surveillance of gastroenteritis, particularly where localised intervention is needed.

School absenteeism data proved to be a representative and timely measure for tracking outbreaks in child-focused settings, benefiting from daily data collection that enables real-time monitoring. Its flexibility allows for application across various infectious diseases, including during large-scale public health events. However, in many cases, symptom information is reported by caregivers and may be submitted voluntarily, which can reduce the overall accuracy and completeness of the data [65]. In addition, the performance of school absenteeism data for gastroenteritis surveillance can be undermined by variability in policy enforcement, where illness exclusion rules are not applied uniformly [65, 74] and implementation [75]. Furthermore, practical constraints on families mean that parents who cannot afford to miss work might still send mildly ill children to school, bypassing exclusion recommendations [21]. These issues reduce the sensitivity and representativeness of absenteeism data, reinforcing the need for standardised reporting practices to improve data reliability in syndromic surveillance systems.

Our scoping review also revealed that a prospective evaluation of the syndromic surveillance system is needed. Most studies conducted the evaluation retrospectively, which may not provide us with the true picture of the reported outcomes. While retrospective evaluations remain a common approach because of their flexibility, cost-effectiveness and ability to establish historical baselines for an infection [11, 68], there is a strong need for more prospective evaluations of syndromic surveillance systems to provide a more comprehensive and realistic assessment of their performance and utility in real-world settings. This recommendation ensures that the approach to surveillance evaluated in a study can be reproduced in public health practice [49], allowing comprehensive assessment of sensitivity, timeliness and other performance attributes within the context of active public health practice.

Additionally, prospective evaluations provide insights into how public health officials interact with the system, making it easier to identify practical challenges and opportunities for improvement [40, 44]. All included studies reported that syndromic surveillance systems were capable of providing early warning signals, potentially enabling public health authorities to respond more rapidly than they could using a traditional surveillance system alone. However, most studies did not describe whether these signals were used in real time to inform public health decision making or outbreak response. The retrospective nature of the studies limits insight into whether early signals were acted upon or led to improved health outcomes. Only one study [65] reported the potential for prospective use to support prevention, reporting that their school absenteeism system (N-SASSy) triggered 85 public health calls, including 70 related to gastroenteritis, which reportedly prevented 67 outbreak clusters. This gap highlights the need for future prospective evaluations that not only assess system performance attributes (e.g., sensitivity, timeliness) but also evaluate system usefulness, particularly in terms of how syndromic surveillance informs real-time decisions and response activities in public health practice.

This review also highlights a lack of comprehensive evaluation of key syndromic surveillance system performance attributes. Most studies focused on sensitivity and timeliness, with limited assessments of other critical attributes such as representativeness, acceptability, and stability. While this can be logical given that syndromic surveillance purpose is to enhance traditional surveillance systems by the reliance on health-related data which is by nature non-diagnostic data to detect abnormal trends in a timely manner [40], these attributes are interconnected [31], makes a comprehensive understanding of these attributes crucial to appropriately understanding a system’s effectiveness.

For example, a critical finding from our review is the impact of representativeness and geographic alignment on the sensitivity and effectiveness of syndromic surveillance systems. For instance, one study observed no clear correlation between national pharmacy data and local outbreak trends in the province of interest [60], suggesting that using national-level data to monitor localised outbreaks may result in missed signals due to inadequate geographic representativeness. Another study found that web traffic in one county was insufficient for detecting outbreaks occurring in other regions [62]. These findings align with another study that reported challenges in detecting localised outbreaks using national syndromic surveillance data [67], further highlighting the need for data that aligns with the geographic and demographic characteristics of the monitored population. In addition, a study conducted by Colón-González et al. [68] supports this finding, arguing that data must be both geographically and demographically representative to ensure effective outbreak detection. Together, these studies underline the importance of ensuring that the data used in syndromic surveillance systems are appropriately representative to maximise sensitivity and timely detection of outbreaks.

The scoping review also indicated that all included studies focus on data analysis outcomes reported by other studies or data availability for their rationale of selecting the data type. While learning from other experiences is the nature of gaining knowledge and improvement, and the availability of the data is not only logical but also helps to ease the collection of the data, particularly with the availability of a structural source as highlighted earlier in the reported finding, none of the studies considered health-seeking behaviour of the population under surveillance as a rationale for data selection. Syndromic surveillance relies on non-diagnostic data that can be classified based on its potential timeliness into pre-clinical data, which provides evidence of illness before an individual seeks health care, such as over-the-counter medication sales and clinical pre-diagnostic data, which precedes a confirmation test after seeking health care, such as emergency department records [11, 42]. It can even be categorised broadly into behavioural indicators, which include data sources tied to the actions of individuals in response to health concerns (e.g., buying medications) and clinical indicators, which include data sources tied to formal healthcare activities (e.g., ordering lab tests, categorising patient complaints) [44]. Understanding these generated actions is crucial for the performance of syndromic surveillance. According to Colón-González et al. [68], when more people use a particular health service, the time to detection will decrease, which means that the infection will be detected faster, indicating timeliness. This is why future research should carefully address the health-seeking behaviour of the targeted population to improve the performance of their syndromic surveillance.

To address this gap, we suggest that future research adopt an integrated approach that combines health service utilisation indicators that do not require in-person health care visit with behavioural frameworks to inform the selection of appropriate data sources for syndromic surveillance. High-use services are likely to produce more timely and representative data for early detection of infectious diseases [68]. Therefore, monitoring the health seeking behaviour such as online search, OTC sales, telehealth calls, and absenteeism can offer useful insights into which services people rely on most which, could help the selection of timely and representative surveillance signals. This can be done by routinely monitoring published government reports that track service utilisation within the same setting or country [76, 77], alongside targeted surveys that explore how people seek care across different communities and demographic groups [78–80]. To complement this, future research could benefit from applying behavioural frameworks to systematically examine how population behaviours influence the utility and representativeness of syndromic surveillance data. Programs grounded in behavioural theory are generally more likely to achieve meaningful outcomes. For example, theoretical models such as the Health Belief Model (HBM) are essential for effective program planning, implementation, and evaluation [81, 82]. This model has been widely applied to predict health behaviours across various diseases and contexts, including vaccination and medication use [83, 84]. Exploring these behavioural insights could support more informed decisions about data source selection, ultimately it could enhance the performance of syndromic surveillance systems in terms of timeliness, representativeness, and overall public health value.

### Strengths

A key strength of this review was the use of the CDC framework for evaluating surveillance systems to systematically guide the extraction of outcomes related to different data types. This approach ensured consistency and comprehensiveness by aligning the analysis with established performance attributes.

#### Limitations

The review mainly focused on the reported outcomes related to the data type utilised in the syndromic surveillance system; however, other elements, like the detection method, may have also impacted these outcomes. It is important to note that this review only considered publications in English from high-income countries, which could mean missing out on perspectives from studies conducted in different languages and regions, leading to potential publication bias. Moreover, the rationale for selecting the data was based on judgment rather than following a structured evaluation process. This may lead to bias when interpreting the studies that were included.

## Conclusion

This scoping review outlines several important considerations for designing or refining syndromic surveillance systems aimed at detecting infectious gastroenteritis outbreaks in HICs. For conditions such as gastroenteritis, which are often self-limiting, non-diagnostic data sources, including over-the-counter medication sales, web search trends, absenteeism records, and telehealth consultations, may offer earlier and potentially more representative signals than data derived from healthcare attendance. The usefulness of these sources, however, depends on how well they match the population characteristics and the specific goals of the surveillance system.

During system design, it is important to consider the health-seeking behaviours of the target population, as these directly influence data reliability and uptake. Overlooking these behaviours can result in missed signals, even in otherwise well-designed systems. In addition, the review also found substantial variation across studies in how data were collected, which symptoms were monitored, and which detection methods were used. This further emphasises caution when applying existing approaches to be adapted to suit the specific population and surveillance objectives within a given context.

Furthermore, more systematic and prospective evaluations to evaluate syndromic surveillance are needed. This review revealed that most studies focused narrowly on sensitivity and timeliness, with limited focus on other essential performance attributes or real-time public health response. A comprehensive evaluation of surveillance system performance beyond sensitivity and timeliness would support a more meaningful understanding of the effectiveness and ensure that systems meet their intended objectives.

By considering these factors during the system development process, we hope that future syndromic surveillance systems may be better positioned to provide timely, sensitive and representative outcomes that enhance public health responses to gastroenteritis outbreaks.

## Supplementary Information

Additional file 1: Table S1. Database search strategy and results.

Additional file 2: Table A. The Primary Purpose of the Study of the Included Studies.

## Data Availability

The data used for this study are included in the main manuscript and in the supplementary files.
