# Supplementary material for: Syndromic surveillance systems to detect outbreaks of gastroenteritis in high-income countries: a scoping review
Source: BMC Public Health. 2026 Mar 23;26:943. doi: 10.1186/s12889-025-25329-w (PMC13011730; doi:10.1186/s12889-025-25329-w)
Supplement: Supplementary file 1 — Additional file 1: Table S1. Database search strategy and results. [file 12889_2025_25329_MOESM1_ESM.docx]

**Appendix**

***Table A. The Primary Purpose of the Study of the Included Studies***

| Author (Year) | Purpose of the Study |
| --- | --- |
| Hogan et al. (2003) | To evaluate whether sales of electrolyte products can serve as an early signal of outbreaks of respiratory and diarrheal diseases in children. |
| Edge et al. (2004) | To evaluate whether monitoring over-the-counter (OTC) drug sales can serve as an effective and timely syndromic surveillance method for detecting outbreaks of gastrointestinal illness. |
| Caudle et al. (2009) | To evaluate whether monitoring Telehealth Ontario can serve as an effective and timely syndromic surveillance method for detecting outbreaks of gastrointestinal illness. |
| Andersson et al. (2014) | To evaluate the effectiveness of different data sources in detecting local outbreaks of acute gastroenteritis. |
| Edelstein et al. (2014) | To evaluate the added value of Websök, an internet-based surveillance system for early disease outbreak detection and seasonality. |
| Pivette et al. (2014) | To evaluate the use of drug sales data (both prescribed and over-the-counter) as a syndromic surveillance tool for the early detection of gastrointestinal (GI) epidemics at the national and regional level. |
| Muchaal et al. (2015) | To evaluate the timeliness, accuracy, and utility of pharmacy-based syndromic surveillance systems in detecting and monitoring both respiratory and gastrointestinal (GI) outbreaks. |
| Tanabe et al. (2019) | To evaluate the effectiveness of the (Nursery) School Absenteeism Surveillance System (N)SASSy in the early detection of gastroenteritis outbreaks by monitoring absenteeism due to symptoms related to gastroenteritis. |
| Hughes et al. (2019) | To evaluate the potential of using telehealth syndromic surveillance data, specifically related to vomiting calls, as an early warning system for detecting seasonal norovirus activity. |
| Donaldson et al. (2022) | To evaluate whether norovirus outbreaks in children could serve as an early warning of seasonal norovirus and outbreaks in the general population. |
