# Supplementary material for: Syndromic surveillance systems to detect outbreaks of gastroenteritis in high-income countries: a scoping review
Source: BMC Public Health. 2026 Mar 23;26:943. doi: 10.1186/s12889-025-25329-w (PMC13011730; doi:10.1186/s12889-025-25329-w)
Supplement: Supplementary file 2 — Additional file 2: Table A. The Primary Purpose of the Study of the Included Studies. [file 12889_2025_25329_MOESM2_ESM.docx]

***Table S1. Database search strategy and results***

| Database | Search string* | Results |
| --- | --- | --- |
| Pubmed | ((((((((((((((((((Syndromic surveillance[MeSH Terms]) OR (Population surveillance[MeSH Terms])) OR (Public Health Surveillance[MeSH Terms])) OR (Sentinel Surveillance[MeSH Terms])) OR (Infodemiology[MeSH Terms])) OR (syndromic surveillance[Title/Abstract])) OR (Early warning systems[Title/Abstract])) OR (Prodrome surveillance[Title/Abstract])) OR (Outbreak detection systems[Title/Abstract])) OR (Information system-based sentinel surveillance[Title/Abstract])) OR (Biosurveillance systems[Title/Abstract])) OR (Health indicator surveillance[Title/Abstract])) OR (Symptom-based surveillance[Title/Abstract]))) OR (Surveillance[Title/Abstract])) OR (Infoveillance[Title/Abstract]))) AND (((((((((((((((((((((((((Emergency Department[MeSH Terms]) OR (Over the counter drugs[MeSH Terms])) OR (Telehealth[MeSH Terms])) OR (Electronic Health Records[MeSH Terms])) OR (Mobile app[MeSH Terms])) OR (Internet-Based Intervention[MeSH Terms])) OR (Cell phone[MeSH Terms])) OR (Data source[MeSH Terms])) OR (Chief Complaint Data[Title/Abstract])) OR (School absenteeism[Title/Abstract])) OR (Outpatient Clinic Data[Title/Abstract])) OR (Ambulance dispatch data[Title/Abstract])) OR (Insurance, Health[MeSH Terms])) OR (Emergency Department[Title/Abstract])) OR (Over the counter drugs[Title/Abstract])) OR (Telehealth[Title/Abstract])) OR (Electronic Health Records[Title/Abstract])) OR (Mobile app[Title/Abstract])) OR (Internet-Based Intervention[Title/Abstract])) OR (Cell phone[Title/Abstract])) OR (Data source[Title/Abstract])) OR (Medical Records Systems, Computerized[MeSH Terms])) OR (Emergency Service, Hospital[MeSH Terms])) OR (Nonprescription Drugs[MeSH Terms])))) AND ((((((gastroenteritis[MeSH Terms]) OR (Diarrhea[MeSH Terms])) OR (vomiting[MeSH Terms])) OR (Gastro*[Title/Abstract])) OR (Diarrhea[Title/Abstract]) OR (vomiting[Title/Abstract])) OR ((((((((((((((((((((((((Norovirus[MeSH Terms]) OR (Rotavirus[MeSH Terms])) OR (Adenoviridae Infections[MeSH Terms])) OR (Astroviridae[MeSH Terms])) OR (Sapovirus[MeSH Terms])) OR (Salmonella[MeSH Terms])) OR (Campylobacter[MeSH Terms])) OR (Escherichia coli[MeSH Terms])) OR (Shigella[MeSH Terms])) OR (Giardia[MeSH Terms])) OR (Cryptosporidium[MeSH Terms])) OR (Entamoeba histolytica[MeSH Terms])) OR (Norovirus[Title/Abstract])) OR (Rotavirus[Title/Abstract])) OR (Adenovirus[Title/Abstract])) OR (Astrovirus[Title/Abstract])) OR (Sapovirus[Title/Abstract])) OR (Salmonella[Title/Abstract])) OR (Campylobact*[Title/Abstract])) OR (Escherichia coli[Title/Abstract])) OR (Shigella[Title/Abstract])) OR (Giardi*[Title/Abstract])) OR (Cryptosporidi*[Title/Abstract])) OR (Entamoeba histolytica[Title/Abstract]))) NOT ((animals[MeSH Terms]) NOT (humans[MeSH Terms])) Filters: from 2000 - 2024/9/12 | 1006 |
| Embase | ('disease surveillance'/exp OR 'disease surveillance' OR 'population surveillance'/exp OR 'population surveillance' OR 'public health surveillance'/exp OR 'public health surveillance' OR 'sentinel surveillance'/exp OR 'sentinel surveillance' OR 'infodemiology'/exp OR 'infodemiology' OR 'syndromic surveillance':ti,ab OR 'early warning systems':ti,ab OR 'prodrome surveillance':ti,ab OR 'outbreak detection systems':ti,ab OR 'information system-based sentinel surveillance':ti,ab OR 'biosurveillance systems':ti,ab OR 'health indicator surveillance':ti,ab OR 'symptom-based surveillance':ti,ab OR surveillance:ti,ab OR infoveillance:ti,ab) AND ('emergency ward'/exp OR 'emergency ward' OR 'non prescription drug'/exp OR 'non prescription drug' OR 'telehealth'/exp OR 'telehealth' OR 'electronic health record'/exp OR 'electronic health record' OR 'mobile application'/exp OR 'mobile application' OR 'web-based intervention'/exp OR 'web-based intervention' OR 'mobile phone'/exp OR 'mobile phone' OR 'data source'/exp OR 'data source' OR 'chief complaint data':ti,ab OR 'school absenteeism':ti,ab OR 'outpatient clinic data':ti,ab OR 'ambulance dispatch data':ti,ab OR 'health insurance'/exp OR 'health insurance' OR 'emergency department':ti,ab OR 'over the counter drugs':ti,ab OR telehealth:ti,ab OR 'electronic health records':ti,ab OR 'mobile app':ti,ab OR 'internet-based intervention':ti,ab OR 'cell phone':ti,ab OR 'data source':ti,ab OR 'electronic medical record system'/exp OR 'electronic medical record system' OR 'hospital emergency service'/exp OR 'hospital emergency service') AND ('gastroenteritis'/exp OR 'gastroenteritis' OR 'diarrhea'/exp OR 'diarrhea' OR 'vomiting'/exp OR 'vomiting' OR gastro*:ti,ab OR diarrhea:ti,ab OR vomiting:ti,ab OR 'norovirus infection'/exp OR 'norovirus infection' OR 'rotavirus infection'/exp OR 'rotavirus infection' OR 'adenovirus infection'/exp OR 'adenovirus infection' OR 'astrovirus infection'/exp OR 'astrovirus infection' OR 'sapovirus'/exp OR 'sapovirus' OR 'salmonella enterica'/exp OR 'salmonella enterica' OR 'campylobacter'/exp OR 'campylobacter' OR 'escherichia coli infection'/exp OR 'escherichia coli infection' OR 'shigellosis'/exp OR 'shigellosis' OR 'giardiasis'/exp OR 'giardiasis' OR 'cryptosporidiosis'/exp OR 'cryptosporidiosis' OR 'amebiasis'/exp OR 'amebiasis' OR norovirus:ti,ab OR rotavirus:ti,ab OR adenovirus:ti,ab OR astrovirus:ti,ab OR sapovirus:ti,ab OR salmonella:ti,ab OR campylobact*:ti,ab OR 'escherichia coli':ti,ab OR shigella:ti,ab OR giardi*:ti,ab OR cryptosporidi*:ti,ab OR 'entamoeba histolytica':ti,ab) NOT (('animal'/exp OR 'animal') NOT ('human'/exp OR 'human')) AND [embase]/lim AND ([article]/lim OR [article in press]/lim OR [review]/lim) AND [2000-2023]/py | 805 |
| Scopus | ( INDEXTERMS ( "Syndromic surveillance" ) OR INDEXTERMS ( "Population surveillance" ) OR INDEXTERMS ( "Public Health Surveillance" ) OR INDEXTERMS ( "Sentinel Surveillance" ) OR INDEXTERMS ( infodemiology ) OR TITLE-ABS ( "syndromic surveillance" ) OR TITLE-ABS ( "Early warning systems" ) OR TITLE-ABS ( "Prodrome surveillance" ) OR TITLE-ABS ( "Outbreak detection systems" ) OR TITLE-ABS ( "Information system-based sentinel surveillance" ) OR TITLE-ABS ( "Biosurveillance systems" ) OR TITLE-ABS ( "Health indicator surveillance" ) OR TITLE-ABS ( "Symptom-based surveillance" ) OR TITLE-ABS ( surveillance ) OR TITLE-ABS ( infoveillance ) ) AND ( INDEXTERMS ( "Emergency Department" ) OR INDEXTERMS ( "Over the counter drugs" ) OR INDEXTERMS ( telehealth ) OR INDEXTERMS ( "Electronic Health Records" ) OR INDEXTERMS ( "Mobile app" ) OR INDEXTERMS ( "Internet-Based Intervention" ) OR INDEXTERMS ( "Cell phone" ) OR INDEXTERMS ( "Data source" ) OR TITLE-ABS ( "Chief Complaint Data" ) OR TITLE-ABS ( "School absenteeism" ) OR TITLE-ABS ( "Outpatient Clinic Data" ) OR TITLE-ABS ( "Ambulance dispatch data" ) OR INDEXTERMS ( "Insurance, Health" ) OR TITLE-ABS ( "Emergency Department" ) OR TITLE-ABS ( "Over the counter drugs" ) OR TITLE-ABS ( telehealth ) OR TITLE-ABS ( "Electronic Health Records" ) OR TITLE-ABS ( "Mobile app" ) OR TITLE-ABS ( "Internet-Based Intervention" ) OR TITLE-ABS ( "Cell phone" ) OR TITLE-ABS ( "Data source" ) OR INDEXTERMS ( "Medical Records Systems, Computerized" ) OR INDEXTERMS ( "Emergency Service, Hospital" ) OR INDEXTERMS ( "Nonprescription Drugs" ) ) AND ( INDEXTERMS ( gastroenteritis ) OR INDEXTERMS ( diarrhea ) OR INDEXTERMS ( vomiting ) OR TITLE-ABS ( gastro* ) OR TITLE-ABS ( diarrhea ) OR TITLE-ABS ( vomiting ) ) OR ( INDEXTERMS ( norovirus ) OR INDEXTERMS ( rotavirus ) OR INDEXTERMS ( "Adenoviridae Infections" ) OR INDEXTERMS ( astroviridae ) OR INDEXTERMS ( sapovirus ) OR INDEXTERMS ( salmonella ) OR INDEXTERMS ( campylobacter ) OR INDEXTERMS ( "Escherichia coli" ) OR INDEXTERMS ( shigella ) OR INDEXTERMS ( giardia ) OR INDEXTERMS ( cryptosporidium ) OR INDEXTERMS ( "Entamoeba histolytica" ) OR TITLE-ABS ( norovirus ) OR TITLE-ABS ( rotavirus ) OR TITLE-ABS ( adenovirus ) OR TITLE-ABS ( astrovirus ) OR TITLE-ABS ( sapovirus ) OR TITLE-ABS ( salmonella ) OR TITLE-ABS ( campylobact* ) OR TITLE-ABS ( "Escherichia coli" ) OR TITLE-ABS ( shigella ) OR TITLE-ABS ( giardi* ) OR TITLE-ABS ( cryptosporidi* ) OR TITLE-ABS ( "Entamoeba histolytica" ) ) AND NOT ( INDEXTERMS ( animals ) not INDEXTERMS ( humans ) ) AND PUBYEAR > 1999 AND PUBYEAR < 2025 | 674 |
| CINAHL | ((TI syndromic surveillance OR AB syndromic surveillance OR MH disease surveillance OR MH population surveillance) AND ((MH gastroenteritis+) OR (MH Diarrhea) OR (MH vomiting+) OR (TI Gastro* OR AB Gastro*) OR (TI Diarrhea OR AB Diarrhea) OR (TI vomiting OR AB vomiting)) | 1024 |

*A filter was used to limit the search history to be between 2000 and 2024
